# Antibodies Directed against a Peptide Epitope of a *Klebsiella pneumoniae*-Derived Protein Are Present in Ankylosing Spondylitis

**DOI:** 10.1371/journal.pone.0171073

**Published:** 2017-01-30

**Authors:** Antonio Puccetti, Marzia Dolcino, Elisa Tinazzi, Francesca Moretta, Salvatore D’Angelo, Ignazio Olivieri, Claudio Lunardi

**Affiliations:** 1 Immunology Area, Pediatric Hospital Bambino Gesù, Rome, Italy; 2 Department of Medicine, University of Verona, Verona, Italy; 3 Rheumatology Department of Lucania, San Carlo Hospital of Potenza, Potenza, Italy; 4 Rheumatology Department of Lucania, Madonna delle Grazie Hospital of Matera, Matera, Italy; University of East London, UNITED KINGDOM

## Abstract

Ankylosing spondylitis (AS) is a chronic inflammatory arthritis of unknown origin. Its autoimmune origin has been suggested but never proven. Several reports have implicated *Klebsiella pneumoniae* as a triggering or perpetuating factor in AS; however, its role in the disease pathogenesis remains debated. Moreover, despite extensive investigations, a biomarker for AS has not yet been identified. To clarify these issues, we screened a random peptide library with pooled IgGs obtained from 40 patients with AS. A peptide (AS peptide) selected from the library was recognized by serum IgGs from 170 of 200 (85%) patients with AS but not by serum specimens from 100 healthy controls. Interestingly, the AS peptide shows a sequence similarity with several molecules expressed at the fibrocartilaginous sites that are primarily involved in the AS inflammatory process. Moreover, the peptide is highly homologous to a *Klebsiella pneumoniae* dipeptidase (DPP) protein. The antibody affinity purified against the AS peptide recognizes the autoantigens and the DPP protein. Furthermore, serum IgG antibodies against the *Klebsiella* DPP_121-145_ peptide epitope were detected in 190 of 200 patients with AS (95%), 3 of 200 patients with rheumatoid arthritis (1.5%) and only 1 of 100 (1%) patients with psoriatic arthritis. Such reactivity was not detected in healthy control donors. Our results show that antibodies directed against an epitope of a *Klebsiella pneumoniae*-derived protein are present in nearly all patients with AS. In the absence of serological biomarkers for AS, such antibodies may represent a useful tool in the diagnosis of the disease.

## Introduction

Ankylosing spondylitis (AS) is one of the most important forms of chronic inflammatory arthritis and represents the “prototype” of a group of interrelated diseases known as spondyloarthritides (SpA). The disease affects primarily the spine, the sacroiliac joints and the peripheral enthesis and joints [1,2]. However, extra-articular manifestations, such as acute anterior uveitis, psoriasis and inflammatory bowel disease, and mainly Crohn’s disease, may be present in nearly half the patients [3].

The class I surface antigen HLA-B27 is associated with the disease, and although the basis of this association remains unclear [3], it seems that aberrant features of HLA-B27 may be involved in the abnormal behaviour of antigen-presenting cells [4,5]. Genome-wide association studies have identified genes involved in the interleukin-23-interleukin-17 pathway that are able to confer susceptibility to AS [6]. Among the other genes found to be associated with the disease, the gene encoding for the aminopeptidase ERAP1 seems to be particularly interesting since the enzyme trims peptides of the appropriate length for binding to MHC class I molecules as follows: gene polymorphisms encode for variants of ERAP1 with different functional activity and interaction with HLA-B27 [7].

The diagnosis of AS is based on a clinical examination and the exclusion of other types of seronegative arthritis. The medical history, physical examination, blood tests, and imaging of the joints may be used for diagnostic purposes. There is no definitive diagnostic test for AS, and, therefore, the identification of a biochemical or immunological test with a high sensitivity and specificity for the diagnosis and follow-up of patients with AS is an important goal in medicine.

The presence of autoantibodies, which are detectable in many rheumatic diseases, is not a typical feature of AS. Antibodies directed against leukocytes [8], neutrophils [9], and some collagen proteins have been previously described [10]. More recently, Wright C. et al. [11] have shown the presence of multiple autoantibodies directed against proteins expressed in connective and skeletal tissue in approximately 40% of patients with AS using a nucleic acid programmable protein array. Nevertheless, autoantibodies typically associated with AS have not, thus far, been identified.

Another aspect of AS that needs to be clarified is the relationship between *Klebsiella pneumoniae* and the aetiopathogenesis of the disease.

A large body of evidence based on genetic, microbiological, molecular and immunological studies suggests that *Klebsiella pneumoniae* is the main microbial agent implicated in the aetiopathogenesis of AS, as a triggering and/or a perpetuating factor [12–14], possibly through a mechanism of molecular mimicry with self-antigens. However, the precise role played by *Klebsiella pneumoniae* in the disease remains controversial and has not yet been clarified.

We aimed to identify a potential serologic marker that may help in the diagnosis and/or monitoring of disease activity in patients with AS. For this purpose, we have used a molecular biological approach, which has been successfully applied to other immune-mediated diseases [15–18].

## Materials and Methods

### Patients

Serum samples from patients and healthy controls were obtained between January 2005 and November 2013. All samples were stored at −20°C. Blood samples were collected after obtaining written informed consent. The present study was approved by the local ethics committee of the Azienda Ospedaliera Universitaria of Verona, Verona, Italy. We analysed a cohort of 200 patients (165 males and 35 females, mean age: 47±14 years) affected by AS, attending the Unit of Autoimmune Diseases at the University Hospital of Verona and the Rheumatology Department of Lucania, San Carlo Hospital of Potenza and Madonna delle Grazie Hospital of Matera. The diagnosis of AS was assessed following the modified Criteria of New York [19]. The clinical features of the 200 patients were as follows: sacroiliitis or spondylitis was present in all the subjects, and peripheral joint arthritis was present in 63/200 patients. Eye involvement was present in 57/200 subjects, and cardiac involvement was present in 4/200 patients. Crohn’s disease was present in 10/200 patients. The HLA B27 allele was detected in 189/200 patients.

A group of 100 patients (64 males and 36 females, mean age: 57±14 years), affected by psoriatic arthritis (PsA), was also studied. All patients fulfilled the Classification Criteria for Psoriatic Arthritis (CASPAR) criteria for the classification of PsA [20]. Two hundred patients with rheumatoid arthritis (RA) were also included in the study. RA patients fulfilled the American College of Rheumatology classification criteria for RA [21]. All patients were consecutively enrolled, regarding of the disease activity and treatment.

One hundred age- and sex-matched healthy donors served as the control group.

All the investigations were carried out according to the principles expressed in the Helsinki Declaration.

### Peptide Library

The screening procedure for the peptide library has been described in detail elsewhere [15–18]. Briefly, a random dodecamer peptide library, which expresses peptides on the surface of *Escherichia coli*, was screened with pooled purified immunoglobulins obtained from the serum samples of 40 patients with AS, according to the manufacturer's instructions (FliTrx Panning Kit, Invitrogen). Following five rounds of biopanning experiments, the enriched library was grown, and single colonies were induced with tryptophan. Bacteria were then lysed in sample buffer and tested by Western blotting with the pooled immunoglobulin fraction obtained from the patients with AS to identify positive clones. DNA was then extracted and sequenced. A set of 15 out of the 27 peptides derived from the last biopanning round was synthetized and employed in a dissociation-enhanced lanthanide fluorescence immunoassay (DELFIA) to test serum samples from individual patients.

### Peptide Synthesis

All the synthetic peptides, including the AS peptide (RIGHVGARPSRH), the *Klebsiella pneumoniae*-derived proline dipeptidase (DPP) peptide (IGYIGPVPER) and the DPP_121-145_ peptide (AARGNIGYIG PVPERALGLG IAADK), the perlecan (PER) peptide (VGTRPSNH), the ADAMTSL3/punctin 2 (ADM) peptide (GHLGARIQR), the Arf GAP With SH3 Domain, the Ankyrin Repeat And PH Domain (ASAP1) peptide (IGHIEGQPSR) and the irrelevant control peptide (VTLPKDSDVELP), were manually synthesized using the standard method of solid-phase peptide synthesis; this method employs the 9-fluorenylmethoxycarbonyl strategy with minor modifications [22].

### Affinity Purification of Anti-Peptide Antibodies

The peptides (5 mg peptide per gram of dried Sepharose powder) were coupled to Sepharose 4B (Pharmacia) following the manufacturer's instructions. Sera diluted in PBS were applied to the columns. Eluted Igs were dialyzed against PBS. The purity of the preparations was checked by SDS-PAGE followed by silver staining.

### Assessment of Antibody Binding

#### a) DELFIA assay

The DELFIA assay is a time-resolved fluorescence method that is commonly used to study antibody interactions with solid-phase proteins or peptides. The method has been described in detail elsewhere [17]. Briefly, the peptides (20 micrograms per millilitre) were used to coat DELFIA plates (PerkinElmer, Waltham, Ma, USA). The plates were then blocked for 1 hour with a commercially available blocking reagent (PerkinElmer). The sera were diluted in phosphate-buffered saline plus 1% bovine serum albumin (Sigma) and incubated on the plates overnight at 4 to 8°C. The plates were washed 10 times with washing buffer (PerkinElmer), and bound antibodies were detected using a europium-labelled anti-human IgG antiserum (1:500 in diluting buffer, PerkinElmer). Plates were read on a Victor3 instrument (PerkinElmer), and the data were analysed with software supplied along with the DELFIA instrument. Absorbance values higher than the mean (+3 SD) for each serum dilution of the control group were considered positive.

#### b) ELISA assay

The ELISA assay for the binding of antibodies to collagen type I and II was performed using commercially available kits (CHONDREX, Inc., Redmond, WA, USA).

In the ELISA assay, for the detection of anti-fibronectin antibodies, plates (Immulon 2HB Thermo Scientific, Illkirch, France) were coated with 10 μg/ml of human fibronectin (SIGMA, St. Louis, MO, USA), and the tested antibodies were diluted in PBS 1% BSA and incubated overnight at 4°C. Plates were washed 3 times with PBS 1% Tween and one time with PBS alone. Alkaline phosphatase-labelled anti-human IgG antibodies were purchased from Sigma. IgG antibodies to *Klebsiella pneumoniae* were detected by ELISA using a bacterial extract adsorbed on the solid phase as described in detail elsewhere [23]. For the binding to recombinant *Klebsiella pneumoniae*, proline dipeptidase (MyBioSource, San Diego, CA, USA) plates were coated with 20 microgram/ml of recombinant protein in PBS. The test was then performed as described above. The binding to the other two *Klebsiella pneumoniae*-derived proteins was assessed using the two synthetic peptides (LFI and SET peptide) using DELFIA as described above.

#### c) Western Blotting

The antibody binding to the Arf GAP with the SH3 Domain, Ankyrin Repeat and PH Domain (ASAP1) protein was tested using the recombinant ASAP1, (OriGene Inc., Rockville, MD, USA) using an immunoblot assay. The blots were probed with primary antibodies (human anti-peptide antibodies or mouse control monoclonal antibody) followed by either peroxidase-conjugated anti-human immunoglobulin antibodies or mouse IgG antibodies (Sigma) (10 μg per millilitre). A monoclonal antibody against ASAP1 was purchased from OriGene and used as a positive control. A monoclonal antibody directed against beta-actin was used as an equal loading control (Abcam).

The blots were developed using chemiluminescence according to the manufacturer’s instructions (Thermo Scientific).

### Statistical Analysis

We evaluated the sensitivity and specificity of the assay with the use of receiver-operating-characteristic (ROC) curve analysis, which evaluates the area under the curve (AUC) with 95% confidence intervals. Statistical analysis was carried out using SPSS Software, Version 20 (SPSS).

## Results

### Peptide Library

We screened a peptide library with pooled Igs obtained from a group of 40 patients with AS. A set of 13 peptides, out of the 26 peptides derived from the last panning step, was used to screen patients’ sera in a DELFIA assay employing the solid phase peptide. We identified a peptide (AS peptide: RIGHVGARPSRH) that was specifically recognized by IgG of serum samples from 33 of 40 patients with AS (83%) (absorbance (mean ± s.d.): 41.900 ± 12.822 for a serum dilution of 1:100) but not by serum IgG from healthy subjects. The results were then validated in another cohort of 160 patients with AS, who were not included in the group used for the library screening. Anti-AS peptide antibodies were found in 137/160 patients; by combining the two cohorts of patients, we observed that 170/200 (85%) patients’ sera contained IgG antibodies directed against the AS peptide. These data indicate that this peptide sequence (AS peptide) contains an epitope, which is recognized by the sera of most patients with AS.

### Autoantigen Targets in Ankylosing Spondylitis

**Fig 1:**
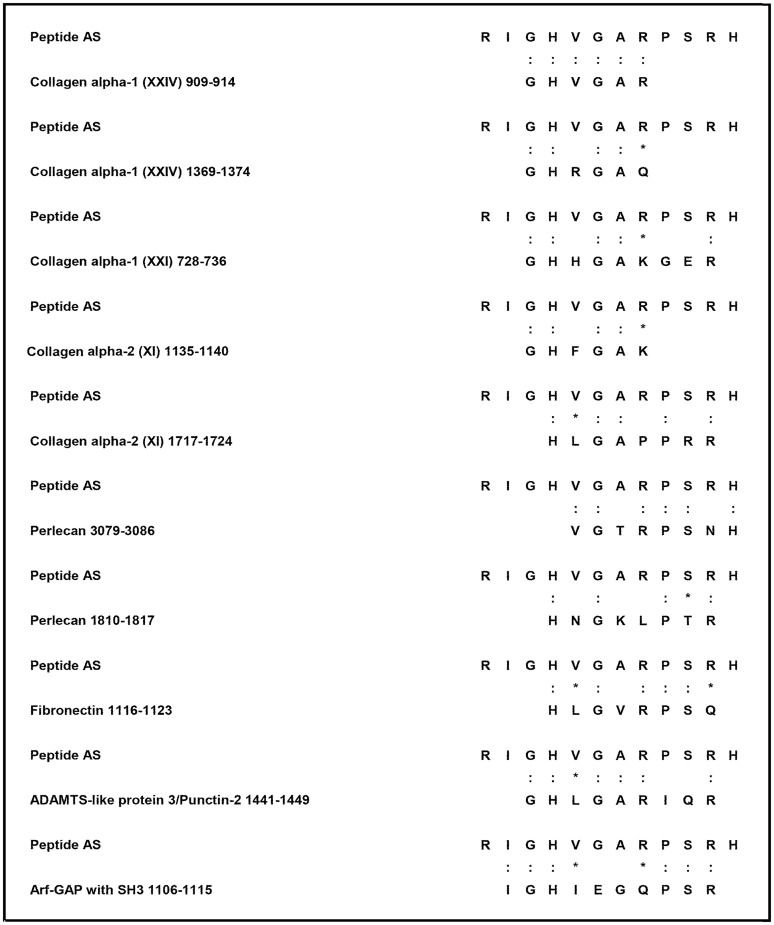
Peptides used in the study and sequence homologies. Sequence homology between the AS peptide and self-proteins (colons indicate identity and asterisks indicate conservative substitutions).

We next compared the AS peptide sequence with human proteins in a protein data bank (SWISS-PROT database of known human sequences), using BLASTP software (NCBI BLAST network service), and we observed that the AS peptide shared homology with different proteins highly expressed at the fibrocartilaginous sites, which are primarily affected in the course of AS. These autoantigens include type I and II collagens (in particular, collagen alpha-1 XXIV [24], collagen alpha-1 XXI [25], and collagen alpha-2 XI, which are particularly abundant at cartilaginous sites [26]), heparan sulphate proteoglycan 2, also known as perlecan, two glycoproteins particularly represented in the extracellular matrix, fibronectin and ADAMTSL3/punctin 2 [27,28], and a protein involved in cytoskeleton remodelling, named ArfGAP with SH3 domain, ankyrin repeat and PH domain 1 actin (ASAP1) [29] (Fig 1).

**Fig 2:**
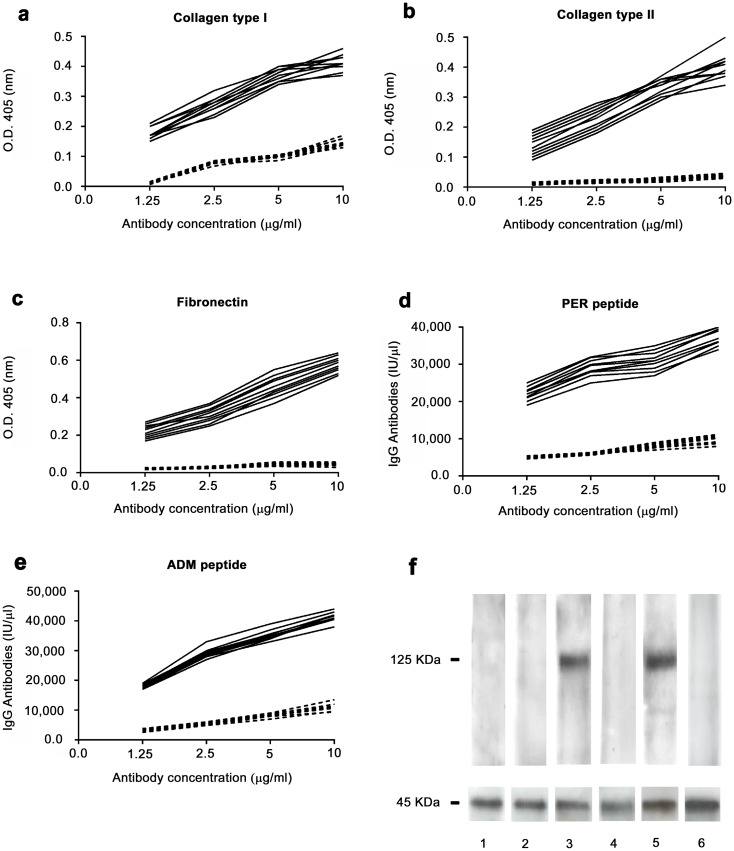
Binding of anti-peptide antibodies to autoantigens. Binding of affinity purified anti-AS peptide antibodies to collagen type I (A), collagen type II (B), fibronectin (C), PER peptide (D), ADM peptide (E). Black continous line: antibodies affinity purified against the AS peptide from the sera of patients affected by AS. Dotted line: antibodies affinity purified against an irrelevant control peptide. A, B and C: ELISA assay. D and E: DELFIA assay. X axis: increasing antibody concentration by two fold ranging from 1.25 microgram/ml to 10 microgram/ml. Y axis: Optical Density values obtained at 405 nm wavelength for ELISA assay and IgG international units for DELFIA assay. F: western blot analysis of the binding of anti-peptide antibodies to ASAP1(molecular weight 125 KDa). Lane 1 and 2, antibodies affinity purified against an irrelevant control peptide probed with cell lysate from cells transfected with ASAP1 (lane 1) or from untransfected cells (lane 2); lane 3 and 4, antibodies affinity purified against the AS peptide probed with cell lysate from cells transfected with ASAP1 (lane 3) or from untransfected cells (lane 4); lanes 5 and 6, commercially available monoclonal antibody directed against ASAP1, probed with cell lysate from cells transfected with ASAP1 (lane 5) or from untransfected cells (lane 6). Lower panel of Fig 2F: immunoblot with actin demonstrates equal protein loading.

Affinity-purified antibodies against the AS peptide isolated from the sera of 10 patients with AS specifically recognized these molecules (Fig 2A–2F). Indeed, anti-AS peptide antibodies bound collagen type I (Fig 2A), collagen type II (Fig 2B) and fibronectin (Fig 2C) in ELISA.

Since the recombinant version of human perlecan and ADAMTSL3/punctin 2 molecules was not available, the cross-reaction of anti-AS peptide antibodies with these proteins was assessed with a DELFIA assay, in which the synthetic peptides (PER and ADM peptides, see above) had been used, as solid phase antigens to coat the DELFIA plates (Fig 2D and 2E). The binding of anti-AS peptide antibodies to ASAP1 was assessed using a cell line (HEK293T) overexpressing the molecule. Antibodies directed against the AS peptide specifically recognized the ASAP1 molecule; in contrast, affinity- purified antibodies directed against an irrelevant control peptide did not react with the ASAP1 protein (Fig 2F). Altogether, these results show that sera of patients with AS contain IgG antibodies able to cross-react with several proteins expressed at the fibrocartilaginous sites that are preferential targets of inflammatory aggression in AS.

### *Klebsiella pneumoniae* and Ankylosing Spondylitis

**Fig 3:**
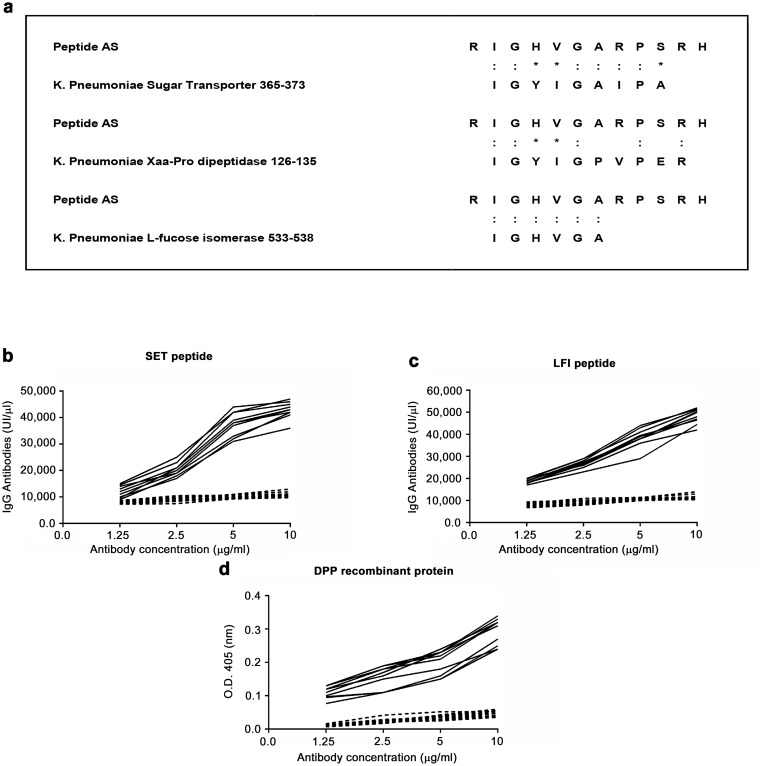
Anti- peptide antibodies bind *Klebsiella pneumoniae*-derived proteins. (A) Sequence homology between the AS peptide and the *Klebsiella pneumoniae*-derived proteins: sugar transporter (SET) protein, Xaa-Pro dipeptidase (DPP) protein and L-fucose isomerase (LFI) protein. Binding of affinity purified anti-AS peptide antibodies (black continuous line) to the SET (B) and LFI (C) peptide in DELFIA. Binding of affinity purified anti-AS peptide antibodies to recombinant DPP protein in ELISA (D). Dotted line: antibodies affinity purified against an irrelevant control peptide. X axis: increasing antibody concentration by two fold ranging from 1.25 microgram/ml to 10 microgram/ml. Y axis: IgG international units for DELFIA assay and Optical Density values obtained at 405 nm wavelength for ELISA assay.

Since *Klebsiella pneumoniae* has been associated with AS [12], we decided to compare the AS peptide sequence with known microbial protein sequences in a protein data bank (SWISS-PROT database), using BLASTP software from the Basic Local Alignment Search Tool (BLAST) network service of the National Center for Biotechnology Information (NCBI). We observed that the AS peptide shares some degree of homology with 3 *Klebsiella pneumoniae*–derived proteins (Fig 3A). Antibodies purified against the AS peptide bound the sugar transporter (SET) peptide (Fig 3B) and the L-fucose peptide (Fig 3C) in DELFIA assay and recognized the recombinant dipeptidase (DPP) protein (Fig 3D). We next used the 3 bacterial peptides in DELFIA to test a panel of 50 sera from AS patients. Antibodies directed against the *Klebsiella pneumoniae* DPP peptide were detected in 36/50 (72%) serum samples; while antibodies against the *Klebsiella pneumoniae* SET and the *Klebsiella pneumoniae* L-fucose isomerase were detected at a lower frequency (37% and 25% of the patients, respectively). Based on these results, we decided to focus our attention on the *Klebsiella pneumoniae*-derived DPP protein. We, therefore, used the recombinant version of the DPP molecule to test a panel of 100 sera from AS patients using a DELFIA assay. Serum IgG antibodies against the *Klebsiella*-derived DPP protein were detected in 88 out of 100 patients with AS, but not in control healthy donors. However, since serum IgG antibodies against the *Klebsiella*-derived peptide (IGYIGPVPER) were detected only in 70 out of 100 patients with AS, we reasoned that the *Klebsiella*-derived DPP protein could contain other crucial epitopes for the anti-*Klebsiella* pneumoniae antibody response.

We, therefore, decided to precisely map the reactivity of AS serum antibodies against the *Klebsiella Pneumoniae-*derived proline dipeptidase protein using 25 amino acids-long synthetic peptides spanning the entire DPP sequence and found that 190 out of the 200 patients with AS had serum IgG antibodies against the peptide DPP_121-145_ (AARGNIGYIGPVPERALGLGIAADK). Interestingly, the ten patients who resulted negative for the presence of anti-DPP_121-145_ IgG antibodies were HLA-B27 negative.

**Fig 4:**
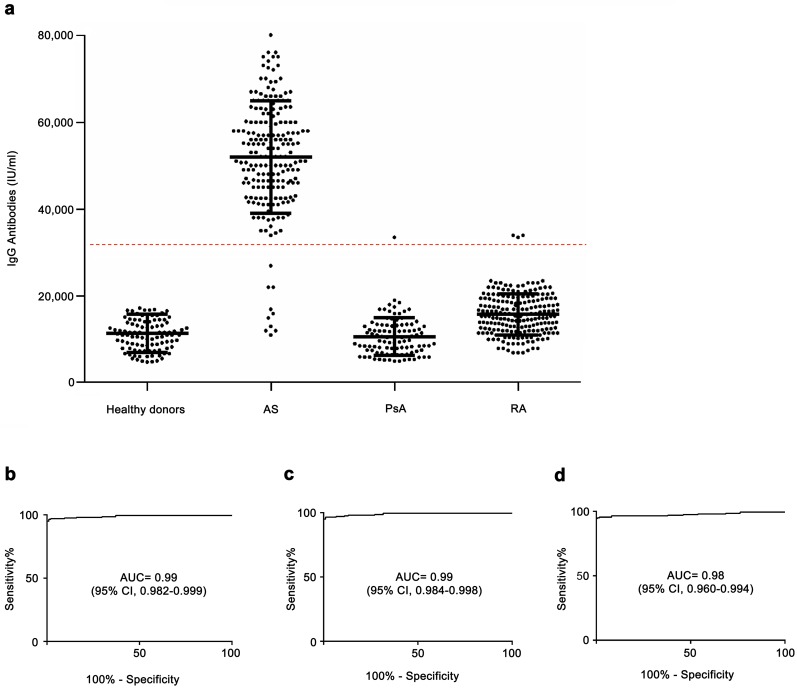
Antibodies directed against the *Klebsiella pneumoniae-*derived DPP_121-145_ peptide in the sera of patients with inflammatory arthritides. (A) The results of an assay of europium-labeled antihuman IgG antibodies are shown; each circle represents a measurement for one patient, and the dashed horizontal line indicates the cut-off value of 32,250 international units. ROC curves showing sensitivity and specificity of the assay of IgG antibodies against the *Klebsiella pneumoniae*-derived DPP_121-145_ peptide for differentiating between AS sera and l healthy donors (B), between AS samples and RA (C) and between AS sera and PsA samples (D).

Anti-peptide antibodies were also investigated in two other inflammatory arthritides, RA and PsA, and these antibodies were present in 3 out of the 200 patients with RA (1.5%) and only in 1 out of the 100 (1%) patients with PsA (Fig 4A).

The sensitivity and specificity of the quantitative analysis of the assay in discriminating AS from healthy donors, with a cut-off value of 32,250 international units, was 95% and 100%, respectively. The area under the curve (AUC) in the ROC analysis was 0.99 (95% confidence interval [Cl] 0.9824 to 0.9990; P<0.0001) (Fig 4B).

The ROC curves obtained, by comparing AS serum specimens with RA and PsA samples, are shown in Fig 4C and 4D. These data show that antibodies directed against a peptide epitope of the *Klebsiella pneumoniae*–derived protein DPP are typically present in the sera of patients with AS.

## Discussion

We report here on a serologic marker that is present in nearly all patients with AS.

In clinical practice, the diagnosis of AS is based on the clinical history, physical examination and imaging, and thus far, there are no biomarkers available to help in the diagnostic process. Moreover, despite extensive investigations, the autoantibodies typically associated with AS and present in the patients’ sera at a high frequency have not been identified thus far, leading to the hypothesis that AS may be an autoinflammatory rather than an autoimmune disease. Indeed, autoantibodies directed against either collagen or neutrophils or nuclear antigens [8–10] have been detected in a few cases, and, in a recent report, which employed a proteomic approach [11], antibodies directed against self-antigens could be found only in 44% of the sera studied.

Using a peptide library approach, we could identify a peptide, the AS peptide, which is specifically recognized by 85% of the sera of patients with AS but is not detectable in the sera of healthy controls.

The peptide shared similarity with different proteins present within the fibrocartilaginous sites that are primarily affected in the course of AS. These autoantigens include type I and II collagens (in particular, collagen alpha-1 XXIV [24], collagen alpha-1 XXI [25], and collagen alpha-2 XI, which is particularly abundant at cartilaginous sites [26]), the heparan sulphate proteoglycan 2, also known as perlecan, two glycoproteins particularly represented in the extracellular matrix, fibronectin and ADAMTSL3/punctin 2 [27,28], and a protein involved in cytoskeleton remodelling, ArfGAP with SH3 domain, ankyrin repeat and PH domain 1 actin (ASAP1) [29].

A possible role for *Klebsiella pneumoniae* in the pathogenesis of AS has been reported [12–14], and although still controversial, this infectious agent is the only one that has been linked with AS. In this study, we found that the AS peptide, which we identified by screening a peptide library with sera from patients with AS, shares similarity with an amino acid sequence of the DPP protein of *Klebsiella pneumoniae*. Interestingly, compared to the frequency of antibodies directed against the AS peptide, antibodies directed against the DPP_121-145_ peptide were detectable at an even higher frequency in AS serum samples (85% versus 95%, respectively). This difference may be related to the length of the DPP_121-145_ peptide that could allow the acquisition of a tridimensional structure.

It is worthwhile mentioning that the patients who did not show antibodies directed against the DPP_121-145_ peptide were HLA-B27 negative, suggesting a relationship between HLA-B27 and the ability to mount an immune response against *Klebsiella pneumoniae*-derived antigens that is cross-reactive with autoantigens. Indeed, the AS peptide has arginine residues that could allow a potential binding to HLA-B27 antigen; it will be of great interest to test the binding of the AS peptide to HLA-B27 antigen for its implication in the previously mentioned cross-reactive immune response.

Moreover, it is noteworthy mentioning that the anti-*Klebsiella pneumoniae*-derived peptide antibodies are nearly absent in sera of patients affected by PsA and RA and are not detected in healthy donors.

Although these results do not clarify the role played by *Klebsiella pneumoniae* in the disease pathogenesis, the ability of purified anti-AS peptide antibodies to recognize both autoantigens that are abundant in the entheses and amino acid sequences of *Klebsiella pneumoniae*-derived proteins suggest a possible link through a molecular mimicry mechanism, as already reported [13]. The presence of antibodies directed against autoantigens in AS raises a question about the role played by such antibodies in the pathogenesis of the disease. Based on the findings in HLA-B27 transgenic rats, where the presence of CD4+ T cells and antigen-presenting cells expressing high levels of HLA-B27 seem to be of critical importance in the pathogenesis of the disease [30], and where a possible pathogenic role seems to be played by non-conventional HLA-B27 molecules [31], we may suggest that such antibodies reflects the presence of an autoimmune process and may be of relevance, as biomarkers of the disease.

We currently aim to evaluate the presence of ERAP1 polymorphisms in the patients studied, and to extend the testing for the presence of identified antibodies in patients from different countries, to confirm the sensitivity and specificity of the test.

In conclusion, we describe here the identification of an antibody reactivity that was detected in the vast majority of patients with AS; we aim at defining whether such reactivity is due to a specific antibody or to different antibodies. In the absence of other disease biomarkers, this antibody reactivity may represent an interesting tool for the diagnosis of AS.
